# Impact of Social Support Ecosystem on Academic Performance of Children From Low-Income Families: A Moderated Mediation Model

**DOI:** 10.3389/fpsyg.2022.710441

**Published:** 2022-04-26

**Authors:** Xiaoqiong Wen, Zhihua Li

**Affiliations:** ^1^College of Education, Hunan Agricultural University, Changsha, China; ^2^Institute of Education, Hunan University of Science and Technology, Xiangtan, China

**Keywords:** social support, dispositional optimism, grit, academic performance, children from low-income families

## Abstract

This study conducted a questionnaire survey involving 513 children from low-income families (mean age = 13.25 ± 2.19 years) to explore the relationship between social support and academic performance as well as the mediating role of dispositional optimism and the moderating role of grit. A structural equation model analysis showed that: (1) social support has a significant positive predictive effect on academic performance and (2) dispositional optimism has a significant mediating effect on the relationship between social support and academic performance. Further, a moderated mediation effect test showed that grit moderates (3) the direct social support effect on academic performance as well as (4) the direct and indirect pathways among social support, dispositional optimism, and academic performance. The results indicate that social support is conducive to the development of dispositional optimism in children from low-income families, thereby improving their academic performance. At the same time, grit can enhance the positive impact of optimism on the academic performance of children from low-income families. This study has important theoretical and practical implications for effectively improving the academic performance of children from low-income families.

## Introduction

The United Nations Children’s Fund defines children living in poverty as those experiencing “deprivation of material, spiritual, and emotional resources needed to survive, develop, and thrive, leaving them unable to enjoy their rights, achieve their full potential, or participate as full and equal members of society” (Bellamy, 2005). China’s contiguous impoverished regions host approximately 40 million children, whose level of development in health and education is significantly lower than the average level (China Children’s Development Foundation, 2018). There is a proverb in Chinese, “all pursuits are of low value; only studying the books is high” (Yu and Suen, 2005). Chinese culture highlights greater educational attainment and academic performance as the main determinant of evenly distributed educational resources (Beaujean et al., 2011). However, children from low-income families have significantly inferior growth environments and development resources, compared to those from more advantaged families. Good academic performance is an important means for children from low-income families to achieve positive development and break the intergenerational poverty cycle (Yoshikawa et al., 2012). However, owing to the burdens of poverty, parents are unable to devote adequate resources to their children’s upbringing, which causes such children to fall behind in their cognitive development, executive functioning, and academic performance, compared to their peers from more advantaged backgrounds (Hair et al., 2015). Based on the main effect model of social support, we see that social support generally benefits individuals’ physiological and psychological process of adapting; it can help individuals resist stress and obtain positive development outcomes (Cohen and Wills, 1985). Both individual and socio-environmental factors impact academic performance (Lee and Shute, 2010). Therefore, this study aims to explore how social factors (social support) and individual factors (dispositional optimism and grit) impact the academic performance of children from low-income families. We hope that this will provide the much needed theoretical basis and empirical support for optimizing their academic performance.

### Social Support Impact on Academic Performance of Children From Low-Income Families

Social support refers to the perceived respect, care, and assistance that a person gains from the social relationships with individuals around them (such as family, friends, and significant others). Social support plays an important role in promoting and maintaining physical and mental health by eliminating or reducing the negative impact of stressful events on individuals (Alloway and Bebbington, 1987). Existing studies have inconsistent views of and conclusions on the impact of social support on the academic performance of children from low-income families. On the one hand, based on the buffering effect model, social support is seen as an important protective resource for individuals facing high-risk or high-stress events (Miloseva et al., 2017). Previous studies have also shown that high levels of social support can positively predict the academic performance of children from families with low socioeconomic statuses (Vasquez et al., 2016).

According to Lerner’s (Lerner and Hood, 1986) developmental contextualism, individual and external environments interact, and personal factors are the closest factors affecting children’s academic performance. The effect of social support on individuals is indirectly affected by individual internal psychological variables (McAdams and Olson, 2010). Based on the self-esteem threat model, social support implies a relationship of inequity between the donor and recipient of aid, which means that acceptance of aid conflicts with values of self-reliance and independence; thus, social support threatens the recipient’s self-worth (Fisher et al., 1982). Moreover, for children from low-income families, continuous high social support levels may cause inertia in the positive perception of their disadvantaged situations, which may hinder their pursuit of academic excellence (Bolger and Amarel, 2007). Previous studies lack in-depth research on the mediating and moderating variables in the above relationship. Therefore, it is necessary to explore the internal mechanisms through which social support impacts the academic performance of children from low-income families.

### The Mediating Role of Dispositional Optimism

Dispositional optimism, which is an important psychological resource, is a long-term, cross-scenario, and stable dispositional tendency to expect good results in the future (Scheier et al., 1986). The conservation of resources theory (COR) states that, when faced with loss of resources, individuals will mobilize resources to cope (Hobfoll, 1989).

According to the COR theory, optimism may be an important manifestation of the mobilization of internal resources by individuals (Alarcon et al., 2013). Children from low-income families have high internal heterogeneity, and some nevertheless possess positive mental abilities (Lever et al., 2005).

Previous studies have found that optimistic individuals often adopt active coping styles when faced with problems and can seek support from a wide range of social networks (Alloway and Bebbington, 1987; Brissette et al., 2002). The positive response, due to optimism, helps children from low-income families maintain positive and optimistic attitudes when faced with pressures and challenges. Moreover, highly optimistic children can view difficulties and setbacks positively and buffer negative learning experiences (Smith and Hoy, 2015).

Previous studies have shown that better social support can effectively enhance an individual’s internal resources (Schöllgen et al., 2011). Therefore, optimism is likely to be the proximal factor that social support has on individual behavior. Siu et al. (2014) found that individuals’ optimistic personality traits are closely related to their academic achievements and can stimulate students’ intrinsic learning motivations. Hence, we chose optimism as a possible mediating variable between social support and academic performance.

### The Moderating Role of Grit

Grit is a non-cognitive ability to persist and remain passionate toward long-term goals, which can motivate individuals to work hard and stick to these goals (Duckworth et al., 2007). Previous studies on academic performance have found that different achievement levels among individuals with equivalent intelligence levels are largely due to their unique grit levels (Duckworth et al., 2007).

Empirical studies have found that individuals with high grit levels can inhibit spontaneous distractions, thereby positively influencing their academic performance (Roux et al., 2015; Liu and Gao, 2020), while individuals with low grit levels use avoidance strategies (such as rejection and giving up), which in turn gives rise to negative expectations toward their future studies (Mosanya, 2019).

However, compared with children from more advantaged families, children from low-income families face more pressure and negative life events. Faced with constantly scarce external resources, children from low-income families need to develop more psychological resources to cope with difficulties (Roux et al., 2015). Kundu (2017) revealed that children from low-income families have greater grit than children from more advantaged families do. Individuals with high grit levels are more persistent and focused when accomplishing long-term learning goals, leading to excellent academic performance.

Previous studies have found that both grit and dispositional optimism can be used as positive individual psychological capital, enabling individuals to have constructive emotional experiences and good quality of will in their studies (Luthans et al., 2006). Therefore, this study proposes that social support affects the academic performance of low-income families through the indirect effect of optimism, and it is speculated that this mediating pathway may be regulated by grit.

## The Present Study

**FIGURE 1:**
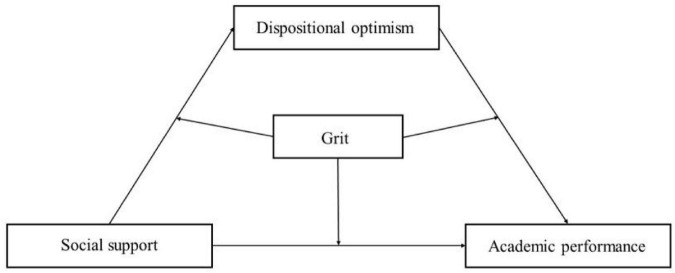
Hypothesized model.

In short, focusing on children from low-income families, this study seeks to explore how external resources (social support) and internal resources (grit and dispositional optimism) impact these children’s’ academic performance. The study proposes the following hypotheses: (1) social support significantly and positively predicts the academic performance of children from low-income families; (2) dispositional optimism mediates between social support and the academic performance of children from low-income families; (3) grit plays a regulating role in the direct and indirect effect pathways of “social support → dispositional optimism → academic performance.” We made use of these hypotheses to build a moderated mediation model which can be seen in Figure 1.

## Materials and Methods

### Participants

The current study used convenience sampling (Creswell, 2007) to select children from nine primary and secondary schools in Hunan Province. The participants were all children from families registered as poverty-stricken, which is the local government’s term for students coming from low-income families, with the family’s monthly income being below 3,000 RMB. Moreover, all of them were required to register poverty according to local standards. With the informed consent of the school, parents, and students, the school screened for students who could fill in the study questionnaires. A total of 527 children (mean age of 13.25 years, standard deviation 2.19) participated in the survey and completed the questionnaires. The number of valid questionnaires collected was 513, with an effective rate of 97.3%; the participants comprised 192 boys (37.5%) and 321 girls (62.5%).

### Measures

#### Social Support

A version of the Child and Adolescent Social Support Rating Scale (CASSS), compiled by Malecki and Demaray (2002) and revised for use in China (Ye and Dai, 2008), was employed in our study. It included 16 items covering three dimensions: subjective support (five items), objective support (six items), and support utilization (five items), graded on a scale of 1 to 5. The sum of all the items’ scores is the total score of the scale, which reflects the overall state of the participant’s social support. The higher the score, the higher the child’s social support level. The Cronbach’s alpha for the social support scale in this study was 0.81.

#### Academic Performance

The participants’ final grades for Chinese, Mathematics, and English were selected as indicators for measuring academic performance. In addition, based on their grades, each participant’s scores were standardized, and the standardized scores of the three subjects were added to obtain the total academic score.

#### Dispositional Optimism

Disposition optimism was measured by the Life Orientation Questionnaire-Revised (LOT-R; Scheier et al., 1994) which includes the two dimensions of optimism and pessimism. The Chinese version of the questionnaire has been proved to have good validity and reliability (Li et al., 2013). A 5-point rating scale was used. The questionnaire included six questions; with questions 1, 3, and 6 testing optimistic expectations, and questions 2, 4, and 5 testing pessimistic expectations. After reverse-coding questions 2, 4, and 5, all items were added to obtain a total score for dispositional optimism. A higher total score indicates that an individual has positive expectations for the future. The Cronbach’s alpha in this study was 0.78.

#### Grit

This study used the revised Chinese version of the Grit-Short scale compiled by Duckworth and Quinn (2009), which included eight items graded on a 5-point rating scale. Among them, questions 1, 3, 5, and 6 were reverse-coded. A higher total score indicates stronger perseverance and tenacity. The Cronbach’s alpha for the scale in this study was 0.79.

### Data Analysis

This study used SPSS 22.0 and PROCESS macro for data processing. The steps involved in the analysis were as follows: (1) Tested the common-method variance and multicollinearity of this study; (2) Compiled descriptive statistics and performed correlation analysis on the variables; and (3) Incorporated gender and age as control variables during the analysis of mediation and moderating effects. Using the structural equation-based mediation and adjustment analysis procedure proposed by Wen and Ye (2014), the study tested the dispositional optimism and grit mediation and moderation effects on the relationship between social support and academic performance.

### Ethics Statement

The study was reviewed and approved by the Ethics Committees of College of Education, Hunan Agricultural University. The study obtained written informed consent to participate in this study from the participants’ legal guardian/next of kin.

## Results

### Common-Method Variance and Multicollinearity Test

An unrotated exploratory factor analysis was performed on all variables, using Harman’s single-factor test (Zhou and Long, 2004). The results indicated that eight factors had characteristic roots greater than one. The variance explained by the first factor was 27.35%, which was lower than the critical value of 40%, indicating that the data have no obvious common-method variance issue. There was a significant correlation between the variables, which may indicate multicollinearity. Therefore, the predictor variables in each equation were standardized (Z score) and collinearity diagnostics were performed. The results indicated that the variance inflation factor for all predictors ranged from 1.15 to 1.24, with a tolerance greater than 0.1. Therefore, it was found that the data had no serious collinearity problem and were thus suitable for further testing.

### Descriptive Statistics and Correlation Analysis for Each Variable

Table 1 lists the mean, standard deviation, and correlation matrix for each variable. As can be seen, the correlation analysis results indicate that gender, age, social support, academic performance, dispositional optimism, and grit are all correlated significantly and positively, meeting the conditions for the moderation effect test (Wen and Ye, 2014). In addition, gender, social support (*t* = 3.26, *p* < 0.01), and academic performance (*t* = 3.25, *p* < 0.01) are all significantly correlated. We also found a significant correlation between age and social support (*t* = 3.87, *p* < 0.001), academic performance (*t* = −2.84, *p* < 0.01), and grit (*t* = 2.82, *p* < 0.01). Therefore, gender and age were used as control variables in subsequent analyses.

**TABLE 1:** Descriptive statistics and correlation matrix for each variable (*n* = 513).

	M ± SD	1	2	3	4	5	6
Gender	0.62 ± 0.48	−					
Age	13.25 ± 2.19	–0.05	−				
Grit	27.15 ± 4.65	–0.04	−0.10*	−			
Dispositional optimism	21.67 ± 3.50	0.02	–0.01	0.33**	−		
Academic performance	0.00 ± 2.52	0.14**	−0.22**	0.20**	0.21**	−	
Social support	61.25 ± 12.34	0.14**	0.18**	0.27**	0.36**	0.13**	−

Gender is a dummy variable (0 = male, 1 = female). * p < 0.05,** p < 0.01,*** p < 0.001 (same as below).

### Testing the Moderated Mediation Model

With gender and age as control variables, the PROCESS macro was used to test the moderated mediation model. The results in Table 2 indicate that social support positively predicts academic performance. The total effect is significant, thus verifying Hypothesis 1. When mediating variables and moderator variables were included, the direct effect of social support on academic performance was not significant; however, social support was found to have had a significant positive predictive effect on temperamental optimism, and temperamental optimism was found to have had a significant positive predictive effect on academic performance. This shows that dispositional optimism plays a mediating role in the social support influence on academic performance, thus verifying Hypothesis 2.

**TABLE 2:** Moderated mediation effect test of social support on academic achievement.

	Equation 1 (dependent variable: academic performance)			Equation 2 (dependent variable: dispositional optimism)			Equation 3 (dependent variable: academic performance)		
	β	*t*	*95% CI*	β	*t*	*95% CI*	β	*t*	*95% CI*
Gender	0.10	2.50	0.12, 0.99	–0.02	–0.31	−0.18, 0.13	0.67	3.04***	0.23, 1.10
Age	–0.25	–5.85	−0.38,−0.19	–0.01	–0.78	−0.05, 0.02	–0.25	−5.19***	−0.35,−0.15
Social support	0.16	3.77***	0.19, 0.63	0.30	7.17***	0.22, 0.39	0.19	1.60	−0.04, 0.42
Grit				0.23	5.53***	0.15, 0.31	0.29	2.55**	0.06, 0.52
Social support and grit				0.09	2.34***	0.01, 0.16	0.21	1.99*	0.01, 0.42
Dispositional optimism							0.40	3.36***	0.16, 0.63
Dispositional optimism * grit							–0.21	−2.0*	−0.42,−0.01
*R* ^2^		0.09			0.20			0.14	
*F*		17.66***			26.14***			12.06***	

The 95% confidence intervals for all predictors were obtained using the bootstrap method. * p < 0.05, ** p < 0.01, *** p < 0.001.

In addition, the interaction term between social support and grit has a significant predictive effect on dispositional optimism, indicating that grit moderates the social support influence on dispositional optimism. In other words, grit moderates the first half of the mediation model. Meanwhile, the interaction term between dispositional optimism and grit has a significant predictive effect on academic performance, which indicates that grit moderates the influence of temperamental optimism on academic performance. In other words, grit adjusts the second half of the mediation model: the interaction between social support and grit has a significant predictive effect on academic performance. This indicates that grit moderates the direct pathway between social support and academic performance, which verifies Hypothesis 3.

**FIGURE 2:**
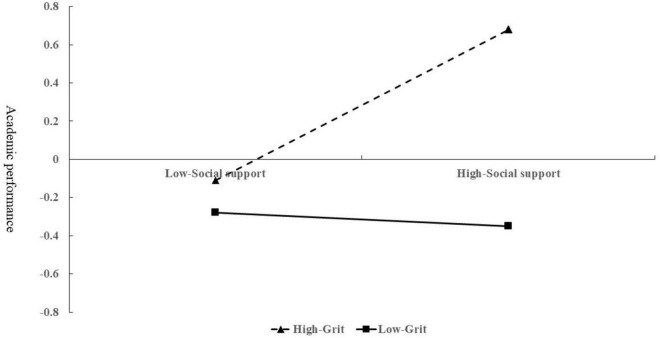
Moderating effect of grit on the relationship between social support and academic performance.

To analyze the trends in its moderating effect further, the aspect of grit was divided into two groups (high and low) to examine its simple effects. The moderating effect of grit on social support and academic performance is shown in Figure 2. Increasing social support levels had no significant impact on the academic performance of children from low-income families with low grit levels (β = 0.02, *t* = 0.14, *p* > 0.05). For children with high grit levels from low-income families, academic performance rapidly increased with an increase in social support levels (β = 0.40, *t* = 2.49, *p* < 0.05).

**FIGURE 3:**
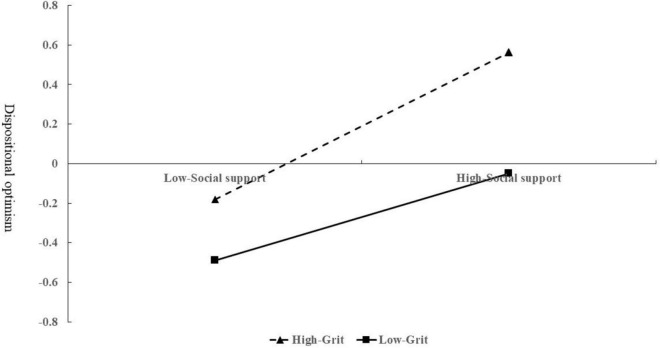
Moderating effect of grit on the relationship between social support and dispositional.

The moderating effect of grit on social support and dispositional optimism is shown in Figure 3. The results indicate that, for children from low-income families with high and low grit levels, higher levels of social support have a certain impact on promoting dispositional optimism (β = 0.21, *t* = 3.70, *p* < 0.001; β = 0.39, *t* = 6.91, *p* < 0.001).

**FIGURE 4:**
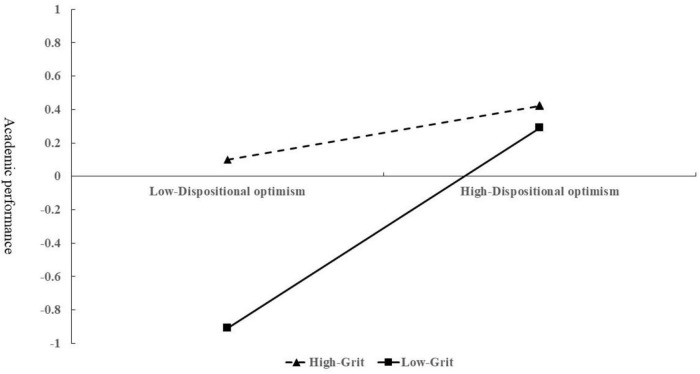
Moderating effect of grit on the relationship between dispositional optimism and academic performance.

The moderating effect of grit on dispositional optimism and academic performance is shown in Figure 4. For children from low-income families with low grit levels, academic performance increased significantly with increased levels of dispositional optimism (β = 0.61, *t* = 3.51, *p* < 0.001). For children from low-income families with high grit levels, academic performance also increased significantly with increased levels of dispositional optimism (β = 0.18, *t* = 1.28, *p* < 0.05).

The study found that grit moderates the process in which social support affects academic performance through dispositional optimism. For children from low-income families with low grit levels, the direct effect of social support on academic performance is weak. For children from low-income families with high or low levels of grit, the social support impact on academic performance through dispositional optimism is stronger.

## Discussion

In this study we created a moderated mediation model, which comprehensively examined the influence of social factors (social support) and individual attributes (dispositional optimism and grit) on academic performance. We explored the moderating effect of dispositional optimism on the relationship between social support and academic performance of children from low-income families, as well as the moderating role of grit in the process. The results revealed that social support mechanisms have certain theoretical and practical significance for improving the academic performance of children from low-income families.

### The Mediating Effect of Dispositional Optimism on Social Support and Academic Performance

Based on the main effect model of social support, it appears that social support benefits individuals’ physiological and psychological adaptation and acts as a protective factor (Cohen and Wills, 1985). The study results indicated that social support has a direct and positive predictive effect on academic performance: higher support levels lead to better academic performance for children from low-income families.

Further analysis found that higher levels of social support improve academic performance by increasing dispositional optimism, which, to a certain extent, corroborates the main effect model. Consistent with the results of previous studies, we found that social support has a positive impact on children from low-income families (Malecki and Demaray, 2006). Our findings underline that individuals with high social support levels have more positive self-perceptions; this includes positive evaluations of their environment and high expectations of future success. The evidence suggests that the emotional warmth provided by social support helps to enhance the optimism of children from low-income families as well (Martínez-Martí and Ruch, 2017).

The results reveal that dispositional optimism helps to improve the academic performance of children from low-income families (Heinonen et al., 2006). Dispositional optimism reflects the positive perception of disadvantaged situations by children from low-income families and their motivational tendency toward successful adaptation. Research outcomes suggest that even if children from low-income families face many difficulties and risk factors, social support and dispositional optimism still play important roles. Therefore, social support may have an impact on their academic performance, through dispositional optimism.

### The Moderating Effect of Grit on the Mediation Effect

The structural equation model analysis shows that grit plays a moderating role in the direct pathway of “social support → dispositional optimism → academic performance.” Consistent with the results of previous studies, it was seen that high grit levels can improve academic performance in children from low-income families (Hagger and Hamilton, 2018). The stress buffer hypothesis states that positive individual factors can play a role in buffering stress, allowing the individual to cope better. As a result, children from low-income families with sound grit attributes can still adapt well in negative situations (Levpuscek et al., 2012; Pereira et al., 2016).

The results also indicate that children from low-income families with high grit levels perform better academically, compared to children from low-income families with low grit levels, regardless of whether they are in environments with high or low social support levels. Previous studies have found that increased social support can motivate children to achieve better academic performance (Rueger et al., 2010). Similarly, when children perceive a lack of social support, they are more driven to change the *status quo* through means such as self-motivated learning and increased resilience (Fan et al., 2018).

The purpose of this study was to show that grit plays a moderating role in the first half of the mediation pathway of “social support → dispositional optimism → academic performance,” indicating that high grit levels play a moderating role in the establishment of dispositional optimism in children from low-income families. Based on the gain and loss spiral model in resource conservation theory, higher grit levels benefit the perception of social support and promote dispositional optimism in children from low-income families (Cao and Qu, 2014).

However, lower grit levels increase the vulnerability of children from low-income families to the pressures and stress caused by resource loss, while the existence of such pressures hinders resource input and accelerates resource loss, thus compounding the problem. This also reveals that a low grit level is not conducive to the development of positive psychological qualities in children from low-income families and that this may lead to a decreased perception of social support. This study also found that grit plays a moderating role in the second half of the mediation pathway of “social support → dispositional optimism → academic performance,” demonstrating that children from low-income families with high grit levels can improve their optimism attributes and thereby maintain good academic performance. Previous studies have shown that the optimism and grit of children from low-income families can buffer academic pressure. Children with higher levels of optimism and grit have stronger internal learning motivations and recognize the value of learning, which positively affects their academic performance (Loftus et al., 2020).

### Implications and Limitations of This Study

In this study we constructed and tested a moderated mediation model and explored the interconnection between social support and academic performance of children from low-income families. Our findings have significant theoretical and practical implications for effectively improving the academic performance of children from low-income families. The results suggest that perceived social support can positively predict the academic performance of children from low-income families. Simultaneously, our findings emphasize the need for improvement of grit levels and dispositional optimism in children from low-income families in order to improve their academic performance.

The study still has some limitations. First, it is difficult for cross-sectional studies to reveal the causal relationships between variables, and we believe that follow-up studies should be designed for future investigations of this topic. Second, the subjective measure (scale) of academic performance can also affect the perception of own performance. We suggest that future studies consider the integration of more objective poverty indicators. Third, this study focused mainly on the protective factors that affect academic performance (social support, grit, and dispositional optimism), and did not explore the adverse factors that affect academic performance. We believe that further investigation as to the effects of these factors is warranted.

## Conclusion

The conclusions of this study are as follows. First, social support has a significant positive predictive effect on the academic performance of children from low-income families. Second, dispositional optimism has a mediating effect on the relationship between social support and academic performance: social support can positively affect the dispositional optimism of children from low-income families, thereby improving their academic performance. Third, grit plays a moderating role in the direct effect of “social support → academic performance,” and in the first and second halves of the indirect effect of “social support → dispositional optimism → academic performance.”

Specifically, social support is conducive to the development of dispositional optimism in children from low-income families, which in turn improves their academic performance. Simultaneously, higher grit can promote the positive impact of dispositional optimism and improvement in academic performance.

## Data Availability Statement

The original contributions presented in the study are included in the article/supplementary material, further inquiries can be directed to the corresponding author/s.

## Ethics Statement

The study was reviewed and approved by the Academic Committee of the College of Education of Hunan Agricultural University. Written informed consent to participate in this study was provided by the participants’ legal guardian/next of kin.

## Author Contributions

XW was mainly responsible for the overall conception and design of this study, and also wrote the manuscript and carried out the statistical analysis. ZL carried out the investigation and data collation work, and the language polishing for the manuscript. Both authors contributed to the article and approved the submitted version.

## Conflict of Interest

The authors declare that the research was conducted in the absence of any commercial or financial relationships that could be construed as a potential conflict of interest.

## Publisher’s Note

All claims expressed in this article are solely those of the authors and do not necessarily represent those of their affiliated organizations, or those of the publisher, the editors and the reviewers. Any product that may be evaluated in this article, or claim that may be made by its manufacturer, is not guaranteed or endorsed by the publisher.

## References

[B1] Alarcon G. M. Bowling N. A. Khazon S. (2013). Great expectations: a meta-analytic examination of optimism and hope. *Pers. Individ. Dif.* 54 821–827. 10.1016/j.paid.2012.12.004

[B44] Alloway R. Bebbington P. (1987). The buffer theory of social support-a review of the literature. *Psychol. Med.* 17, 91–108. 10.1017/S0033291700013015 3575581

[B2] Beaujean A. A. Firmin M. W. Attai S. Johnson C. B. Firmin R. L. Mena K. E. (2011). Using personality and cognitive ability to predict academic achievement in a young adult sample. *Pers. Individ. Dif.* 51 709–714. 10.1016/j.paid.2011.06.023

[B3] Bellamy C. (2005). The state of the world’s children, 2005: childhood under threat. *Future Surv.* 6 71–73. 10.1111/j.1365-2923.1987.tb00517.x 3821604

[B4] Bolger N. Amarel D. (2007). Effects of social support visibility on adjustment to stress: experimental evidence. *J. Pers. Soc. Psychol.* 92:458. 10.1037/0022-3514.92.3.458 17352603

[B5] Brissette I. Scheier M. F. Carver C. S. (2002). The role of optimism in social network development, coping, and psychological adjustment during a life transition. *J. Pers. Soc. Psychol.* 82:102. 10.1037//0022-3514.82.1.102 11811628

[B6] Cao X. Qu J. J. (2014). The origin of resource preservation theory, analysis of main contents and enlightenment. *Hum. Res. Dev. China* 15 75–80.

[B45] China Children’s Development Foundation (2018). *China Child Development Report 2017: Anti Poverty and Early Childhood Development*. Beijing: China Development Press.

[B7] Cohen S. Wills T. A. (1985). Stress, social support, and the buffering hypothesis. *Psychol. Bull.* 98:310. 10.1037/0033-2909.98.2.310 3901065

[B8] Creswell J. W. (2007). in *Qualitative Inquiry and Research Design: Choosing Among Five Approaches*, 2nd Edn, ed. Poth C. N. (London: Sage), 488.

[B9] Duckworth A. L. Peterson C. Matthews M. D. Kelly D. R. (2007). Grit: perseverance and passion for long-term goals. *J. Pers. Soc. Psychol.* 92:1087. 10.1037/0022-3514.92.6.1087 17547490

[B10] Duckworth A. L. Quinn P. D. (2009). Development and validation of the short grit scale (Grit-S). *J. Pers. Assess.* 91 166–174. 10.1080/00223890802634290 19205937

[B11] Fan X. H. Zhou N. He Q. Yu L. Z. Zhu D. Meng H. (2018). Psychological capital and academic achievement of left behind children in rural areas: mediating effect with regulation. *Chinese J. Clin. Psychol.* 3 551–556.

[B12] Fisher J. D. Nadler A. Whitcher-Alagna S. (1982). Recipient reactions to aid. *Psychol. Bull.* 91:27. 10.1037/0033-2909.91.1.27

[B13] Hagger M. S. Hamilton K. (2018). Grit and self-discipline as predictors of effort and academic attainment. *Br. J. Educ. Psychol.* 89 324–342. 10.1111/bjep.12241 30101970

[B14] Hair N. L. Hanson J. L. Wolfe B. L. Pollak S. D. (2015). Association of child poverty, brain development, and academic achievement. *JAMA pediatr.* 169 822–829. 10.1001/jamapediatrics.2015.1475 26192216 PMC4687959

[B15] Heinonen K. Räikkönen K. Matthews K. A. Scheier M. F. Raitakari O. T. Pulkki L. (2006). Socioeconomic status in childhood and adulthood: associations with dispositional optimism and pessimism over a 21-year follow-up. *J. Personal.* 74 1111–1126. 10.1111/j.1467-6494.2006.00404.x 16787430

[B16] Hobfoll S. E. (1989). Conservation of resources: a new attempt at conceptualizing stress. *Am. Psychol.* 44 513–524. 10.1037/0003-066X.44.3.513 2648906

[B17] Kundu A. (2017). Grit and agency: a framework for helping students in poverty to achieve academic greatness. *Natl. Youth-At-Risk J.* 2:69. 10.20429/nyarj.2017.020205

[B18] Lee J. Shute V. J. (2010). Personal and social-contextual factors in K–12 academic performance: an integrative perspective on student learning. *Educ. Psychol.* 45 185–202. 10.1080/00461520.2010.493471

[B46] Lerner R. M. Hood K. E. (1986). Plasticity in development: concepts and issues for intervention. *J. Appl. Dev. Psychol*. 7, 139–152. 10.1016/0193-3973(86)90025-0

[B19] Lever J. P. Pinol N. L. Uralde J. H. (2005). Poverty, psychological resources and subjective well-being. *Soc. Indic. Res.* 73 375–408. 10.1007/s11205-004-1072-7

[B20] Levpuscek M. P. Zupancic M. Socan G. (2012). Predicting achievement in mathematics in adolescent students: the role of individual and social factors. *J. Early Adolesc.* 33 523–551. 10.1177/0272431612450949

[B21] Li Z. Yin X. Y. Cai T. S. (2013). The structure of dispositional optimism: traditional factor models and bifactor model. *Chinese J. Clin. Psychol.* 21 45–48.

[B22] Liu Z. M. Gao W. W. (2020). The relationship between perseverance and academic achievement: different mediating effects of intentional and spontaneous wandering. *Psychol. Sci.* 43 1348–1354.

[B23] Loftus T. J. Filiberto A. C. Rosenthal M. D. Arnaoutakis G. J. Upchurch G. R. Dimick J. B. (2020). Performance advantages for grit and optimism. *Am. J. Surg.* 220 10–18. 10.1016/j.amjsurg.2020.01.057 32098653

[B24] Luthans F. Avey J. B. Avolio B. J. Norman S. M. Combs G. M. (2006). Psychological capital development: toward a micro-intervention. *J. Organ. Behav.* 27 387–393. 10.1002/job.373

[B25] Malecki C. K. Demaray M. K. (2006). Social support as a buffer in the relationship between socioeconomic status and academic performance. *Sch. Psychol. Q.* 21:375. 10.1037/h0084129

[B26] Malecki C. K. Demaray M. K. (2002). Measuring perceived social support: development of the child and adolescent social support scale (CASSS). *Psychol. Sch.* 39 1–18. 10.1002/pits.10004

[B27] Martínez-Martí M. L. Ruch W. (2017). Character strengths predict resilience over and above positive affect, self-efficacy, optimism, social support, self-esteem, and life satisfaction. *J. Posit. Psychol.* 12 110–119. 10.1080/17439760.2016.1163403

[B47] McAdams D. P. Olson B. D. (2010). Personality development: continuity and change over the life course. *Ann. Rev. Psychol*. 61, 517–542. 10.1146/annurev.psych.093008.100507 19534589

[B28] Miloseva L. Vukosavljevic-Gvozden T. Richter K. Milosev V. Niklewski G. (2017). Perceived social support as a moderator between negative life events and depression in adolescence: implications for prediction and targeted prevention. *EPMA J.* 8 237–245. 10.1007/s13167-017-0095-5 29021834 PMC5607153

[B29] Mosanya M. (2019). Exploring cultural intelligence relationships with growth mindset, grit, coping and academic stress in the United Arab Emirates. *Middle East J. Positive Psychol.* 5 42–59.

[B30] Pereira A. Pereira C. R. Monteiro M. B. (2016). Normative pressure to reduce prejudice against homosexuals: the buffering role of beliefs about the nature of homosexuality. *Pers. Individ. Dif.* 96 88–99. 10.1016/j.paid.2016.02.042

[B31] Roux C. Goldsmith K. Bonezzi A. (2015). On the psychology of scarcity: when reminders of resource scarcity promote selfish (and generous) behavior. *J. Consum. Res.* 42 615–631. 10.2139/ssrn.2147919

[B32] Rueger S. Y. Malecki C. K. Demaray M. K. (2010). Relationship between multiple sources of perceived social support and psychological and academic adjustment in early adolescence: comparisons across gender. *J. Youth Adolesc.* 39:47. 10.1007/s10964-008-9368-6 20091216

[B33] Scheier M. F. Carver C. S. Bridges M. W. (1994). Distinguishing optimism from neuroticism (and trait anxiety, self-mastery, and self-esteem): a reevaluation of the Life orientation test. *J. Pers. Soc. Psychol.* 67:1063. 10.1037//0022-3514.67.6.1063 7815302

[B34] Scheier M. F. Weintraub J. K. Carver C. S. (1986). Coping with stress: divergent strategies of optimists and pessimists. *J. Pers. Soc. Psychol.* 51:1257. 10.1037/0022-3514.51.6.1257 3806361

[B35] Schöllgen I. Huxhold O. Schüz B. Tesch-Römer C. (2011). Resources for health: differential effects of optimistic self-beliefs and social support according to socioeconomic status. *Health Psychol.* 30:326. 10.1037/a0022514 21553976

[B36] Siu O. L. Bakker A. B. Jiang X. (2014). Psychological capital among university students: relationships with study engagement and intrinsic motivation. *J. Happiness Stud.* 15 979–994. 10.1007/s10902-013-9459-2

[B37] Smith P. A. Hoy W. K. (2015). Academic optimism and student achievement in urban elementary schools. *J. Educ. Admin.* 45 556–568. 10.1108/09578230710778196

[B38] Vasquez A. C. Patall E. A. Fong C. J. Corrigan A. S. Pine L. (2016). Parent autonomy support, academic achievement, and psychosocial functioning: a meta-analysis of research. *Educ. Psychol. Rev.* 28 605–644. 10.1007/s10648-015-9329-z

[B39] Wen Z. L. Ye B. J. (2014). Moderated mediation model test method: competition or substitution. *J. Psychol.* 46 714–726.

[B40] Ye Y. M. Dai X. Y. (2008). Development of Social Support Scale for university students. *Chinese J. Clin. Psychol.* 16 456–458.

[B41] Yoshikawa H. Aber J. L. Beardslee W. R. (2012). The effects of poverty on the mental, emotional, and behavioral health of children and youth: implications for prevention . *Am. Psychol.* 67:272. 10.1109/98.486976 22583341

[B42] Yu L. Suen H. K. (2005). Historical and contemporary exam-driven education fever in China. *KEDI J. Educ. Policy* 2 17–33.

[B43] Zhou H. Long L. R. (2004). Statistical test and control method of common method deviation. *Prog. Psychol. Sci.* 12 942–942.

